# Blood-Based Biomarkers in Frontotemporal Dementia: A Narrative Review

**DOI:** 10.3390/ijms252111838

**Published:** 2024-11-04

**Authors:** Ioannis Liampas, Panagiota Kyriakoulopoulou, Vasiliki Karakoida, Panagiota Andriana Kavvoura, Markos Sgantzos, Dimitrios P. Bogdanos, Polyxeni Stamati, Efthimios Dardiotis, Vasileios Siokas

**Affiliations:** 1 Department of Neurology, University Hospital of Larissa, School of Medicine, University of Thessaly, 41100 Larissa, Greece; tzeni_0@yahoo.gr (P.S.); edar@med.uth.gr (E.D.); vsiokas@med.uth.gr (V.S.); 2 School of Medicine, University of Patras, 26504 Rio Patras, Greece; panagiotakyriak@yahoo.com (P.K.); bkarakoida@gmail.com (V.K.); nagiakav1999@gmail.com (P.A.K.); 3 Department of Anatomy, Medical School, University of Thessaly, 41100 Larissa, Greece; sgantzos@med.uth.gr; 4 Department of Rheumatology and Clinical Immunology, Faculty of Medicine, School of Health Sciences, University of Thessaly, 41100 Larissa, Greece; bogdanos@med.uth.gr

**Keywords:** neurofilament light chain, phosphorylated neurofilament heavy chain, tau, p-tau, progranulin, glial fibrillary acidic protein, TAR DNA-binding protein-43

## Abstract

This narrative review explores the current landscape of blood biomarkers in Frontotemporal dementia (FTD). Neurofilament light chain (NfL) may be useful in the differentiation of behavioral variant FTD from primary psychiatric disorders (PPDs) or dementia with Lewy bodies (DLB). In prodromal FTD and presymptomatic mutation carriers (*GRN*, *MAPT*, *C9orf72*), elevated NfL may herald pheno-conversion to full-blown dementia. Baseline NfL correlates with steeper neuroanatomical changes and cognitive, behavioral and functional decline, making NfL promising in monitoring disease progression. Phosphorylated neurofilament heavy chain (pNfH) levels have a potential limited role in the demarcation of the conversion stage to full-blown FTD. Combined NfL and pNfH measurements may allow a wider stage stratification. Total tau levels lack applicability in the framework of FTD. p-tau, on the other hand, is of potential value in the discrimination of FTD from Alzheimer’s dementia. Progranulin concentrations could serve the identification of *GRN* mutation carriers. Glial fibrillary acidic protein (GFAP) may assist in the differentiation of PPDs from behavioral variant FTD and the detection of *GRN* mutation carriers (additional research is warranted). Finally, TAR DNA-binding protein-43 (TDP-43) appears to be a promising diagnostic biomarker for FTD. Its potential in distinguishing TDP-43 pathology from other FTD-related pathologies requires further research.

## 1. Introduction

Frontotemporal dementia (FTD) is a holistic term encompassing a spectrum of clinico-pathological entities that involve profound alterations in behavior and/or executive function and/or language and/or motor symptoms that are attributed to progressive focal degeneration of the frontal and/or temporal lobes [1,2,3]. Epidemiological evidence suggests that FTD constitutes the second most prevalent form of young-onset dementia and the third most common neurodegenerative cause of late-onset dementia [1,2]. Its incidence is estimated to be 2–4 new cases per 100,000 per annum with an average age of onset ranging between 45 and 65 years [4]. FTD is equally common in men and women and its prevalence (estimated to be 15–22 per 100,000) peaks during the second half of the 7th decade of life [5]. A salient point of interest is that the true prevalence of this disorder is likely underestimated. Given the diagnostic complexities inherent in FTD, a plethora of psychiatric and neurological conditions can mimic the spectrum of its symptomatology and may lead to misidentification and, in turn, underestimation of its actual frequency [2].

Specific clinical and neuropathological diagnostic criteria are used to differentiate FTD from other pathologies that may also impact frontotemporal structures [6]; nevertheless, the scenery of FTD continuously undergoes remarkable changes requiring constant readaptation [7]. Contemporary investigations show a particularly growing interest in the development of blood-based biomarkers. This approach is becoming increasingly popular considering its minimally invasive procedure (blood sample collection) -opposite to cerebrospinal fluid (CSF)-based markers. Moreover, blood biomarkers offer significant advantages over imaging markers in terms of cost-effectiveness, tolerability (e.g., claustrophobia, exposure to radiation and radioactive tracers; specific preparations may be required for certain investigations), and procedures (imaging examinations are more laborious and time-consuming), making their widespread use possible.

The purpose of this narrative review is to summarize published literature on blood-based biomarkers for FTD, exploring their potential roles in the diagnosis, prognosis, or other important disease aspects. Genetic biomarkers (e.g., genetic mutations, single nucleotide polymorphisms, and circulating RNAs) were intentionally omitted from the current paper; the vast literature on genetic markers warrants a separate investigation from the remaining blood-based markers. Owing to their epidemiologic features and clinical similarities, specific focus was placed on the diagnostic properties of blood biomarkers in the differentiation of FTD from Alzheimer’s disease dementia (AD), dementia with Lewy bodies (DLB), and primary psychiatric disorders (PPDs).

### 1.1. Frontotemporal Dementia: A Brief History

FTD was first described in 1892 by Arnold Pick as a progressive deterioration of language associated with focal left anterior temporal lobe degeneration. Almost 20 years later, Alois Alzheimer conducted histological analysis of Pick’s clinical cases and described ballooned cells and silver-staining argyrophilic cytoplasmic inclusions within neurons [4]. In 1932, Von Braunmuhl identified an association with amyotrophic lateral sclerosis (ALS) for the first time, and in 1936, Grünthal suggested a potential hereditary link while describing the condition in two brothers [8]. In 1957, the French group of Delay, Brion, and Escourolle emphasized the differentiation of FTD from AD, with a specific focus on elucidating its clinical and histopathological characteristics [8]. The landscape of FTD began to resemble the current picture in 1974, when Constantinidis divided Pick’s disease into three subtypes based on clinical and histopathological features [9]. From the last decade of the past century, the first diagnostic criteria for FTD were formulated and later reviewed to keep in pace with the dizzying progress in the fields of neuroimaging and molecular research [6,10,11].

### 1.2. Clinical Spectrum

The distinct clinical subtypes of FTD include the behavioral variant of frontotemporal dementia (bvFTD) and the language variants of primary progressive aphasia (PPA): semantic (svPPA) and nonfluent/agrammatic (nfvPPA), whereas the logopenic variant (lvPPA) is categorized as the most common aphasia phenotype of AD [12,13,14]. bvFTD exhibits early and insidious changes in behavior and personality, such as disinhibition, apathy, lack of empathy, compulsions, hyperorality, and cognitive impairment, particularly in executive function [11,15]. The term PPA is utilized when language-led symptoms emerge gradually in the early disease stages [16,17,18]. In addition, a growing body of evidence supports the notion of clinico-pathological overlap (common and pathological and genetic attributes) between FTD and ALS, also known as motor neuron disease (MND) [19,20].

### 1.3. Genetic Associations

The discovery that a considerable proportion of FTD cases are familial (approximately 10 to 50%, depending on the subtype) was pivotal [21]. In the majority of familial FTD cases, hexanucleotide expansions in the *C9orf72* gene (20–30%), mapped on chromosome 9, autosomal dominant mutations on chromosome 17 involving the granulin (*GRN*) gene (5–25%) and microtubule-associated protein tau (*MAPT*) gene (5–20%) are identified [21,22]. Other genes responsible for less than 5% of cases are *CHMP2B, VCP, TBK1, TIA1, TBP, OPTN, TARDBP, TREM2, UBQLN2, SQSTM1, CCNF, FUS, ITM2B* and *CHCHD10* [21,22,23,24]. Remarkably, sporadic cases of FTD are also due to mutations: *MAPT* 0–2% to *GRN* 5%, and *C9orf72* 6% [22].

### 1.4. Neuropathology

Protein misfolding leading to functional loss and aggregation is considered a pivotal step in the cascade of neurodegeneration [25]. If proteinopenia rather than the formation of insoluble protein piles in the brain constitutes the pivotal detrimental step towards degeneration, it is still under debate [26,27]. In α-Synucleinopathies, α-synuclein agglomerations constitute the main constituent of Lewy bodies and neurites [28]. Amyloid-β and hyperphosphorylated tau deposits primarily in the temporal and parietal lobes are crucial in the cascade of AD-related neurodegeneration [29]. FTD, on the other hand, is characterized by frontotemporal lobar degeneration (FTLD), involving the pathological accumulation of proteins in the frontal and temporal lobes, neuronal loss and astrocytic gliosis [16].

The neuropathology nomenclature of FTD can be categorized into four major patterns depending on the dominant protein accumulation: (1) tau-positive inclusion pathology (FTLD-tau), (2) Tar DNA binding protein 43 (TDP-43) positive inclusion pathology (FTLD-TDP), (3) FET protein family pathology (FTLD-FET), including fused in sarcoma (FUS) protein, (4) immunoreactive for ubiquitin and negative for tau, TDP-43, and FET inclusion pathology (FTLD-UPS) [4]. With respect to tau, it is acknowledged that alternative splicing of the *MAPT* gene generates six different tau isoforms [30]. Imbalances in the TAU isoform ratio can lead to neurodegenerative diseases: neurofibrillary tangles in AD are composed of both hyperphosphorylated 4R tau and 3R tau inclusions; 3R tau aggregates characterize Pick’s disease in FTLD-tau (which is usually sporadic and more rarely due to *MAPT* mutations), whereas 4R tauopathies include corticobasal degeneration (CBD), progressive supranuclear palsy (PSP), globular glial tauopathy (GGT), and argyrophilic grain disease (AGD) [4]. Regarding TDP-43, FTLD-TDP is further subdivided into four subtypes: type A neuropathology is often associated with *GRN* mutations, type B is commonly related to *C9orf72* mutations, type C is the most common neuropathology substrate of svPPA, and type D is uniquely linked to *VCP* gene mutations.

### 1.5. Diagnosis of Frontotemporal Dementia

The diagnosis of FTD necessitates a comprehensive clinical and neuropsychological evaluation and can be supplemented by neuroimaging or genetic analyses. Specific diagnostic criteria emphasize the gradual onset of symptoms affecting behavior, language, and executive function [4]. The clinical criteria for possible bvFTD include behavioral disinhibition, apathy, loss of empathy, perseverative—stereotyped—compulsive behaviors, hyperorality or dietary changes, and executive dysfunction. The clinical diagnosis of svPPA is supported by impaired confrontation naming, single-word comprehension, object knowledge and surface dyslexia or dysgraphia, whereas the clinical diagnosis of nfPPA is based on the presence of agrammatism, effortful—halting—inconsistent speech, and impaired comprehension of syntactically complex sentences.

Imaging findings such as atrophy patterns on magnetic resonance imaging (MRI) and/or computed tomography (CT), hypometabolism or hypoperfusion patterns on positron-emission tomography (PET), or single-photon emission computed tomography (SPECT) scans are capitalized on to increase the probability of an accurate clinical diagnosis (probable or imaging-supported diagnosis). Histopathologic evidence of FTLD on biopsy or postmortem, or the presence of known pathogenic mutations, confirm the diagnosis (definite diagnosis). The clinical diagnosis of FTD often proves challenging due to the overlapping presentation of the disorder with other neurodegenerative entities, such as AD, DLB, or PPDs [1,3]. The need for prompt and accurate diagnosis implies the integration of easy-to-use, cost-compatible, and upgraded diagnostic tools into daily clinical practice.

## 2. Methods

A comprehensive literature search of the MEDLINE (via PubMed) database was performed (last updated on May 2024). The following search strategy was applied: [(biomarkers) AND (frontotemporal dementia)) AND ((blood OR serum OR plasma)] yielding 432 articles. Titles and abstracts were initially screened; in cases of eligibility, full texts were retrieved. Followingly, the literature search was expanded using the Google Scholar search engine. The terms ‘frontotemporal dementia’, ‘biomarkers’ and at least one of the terms ‘blood; serum; plasma’ were utilized as search terms. Google Scholar yielded tens of thousands of articles which were, in turn, sorted by relevancy. Only the first 500 were assessed for eligibility. A manual search including papers cited in published reviews and meta-analyses was finally conducted.

We focused on the retrieval of studies investigating the diagnostic properties of blood biomarkers in the differentiation of FTD from healthy controls (HC), AD, DLB, and PPDs. In case authors grouped together cases with FTD, PSP, or CBD (FTLD spectrum disorders), results for FTLD versus HC, AD, DLB, and PPD were reported. Emphasis was finally placed on the prognostic properties of blood biomarkers (conversion rates, structural changes, cognitive—behavioral—functional decline, survival rates).

## 3. Exploring Blood Biomarkers

A biomarker is commonly defined as an objectively measured indicator of normal biological processes, pathogenic processes, or pharmacologic responses to a therapeutic intervention. Biomarkers play vital roles in various aspects of medicine, including establishing the correct diagnosis, determining prognosis, monitoring treatment responses, and developing new drugs. They provide valuable insights into the underlying biological processes of diseases and aid in personalized medicine approaches. Examples of widely used biomarkers in dementia are brain imaging (CT, MRI, PET, SPECT) and CSF biomarkers [16]. The potential development of blood-based biomarkers garners exceptional interest within the scientific community since they stand for a less invasive (lumbar puncture not required) and hopefully cost-effective approach [31,32]. By harnessing the selective communication between the CSF and the systemic circulation via the blood–brain barrier (BBB), through which proteins that amass within the brain ultimately leak into the bloodstream, researchers are uncovering potential blood biomarkers in dementia research [16]. In this article, we will review some of the most rigorously examined blood biomarkers [measured in serum (after blood clot), plasma (addition of anticoagulant), or total blood] in the field of FTD, discussing their implications in clinical practice (mainly diagnosis and prognosis) (Table 1). ELISA (Enzyme-Linked Immunosorbent Assay), ECL (Electrochemiluminescence), and Simoa (Single Molecule Array) are the most common approaches for measuring blood biomarker levels, owing to their analytical sensitivity. Among these techniques, Simoa seems to be more attractive due to its higher sensitivity and superior correlation with CSF levels; it can detect proteins in blood at sub-femtomolar concentrations (i.e., 10^−16^ M), compared to traditional immunoassays that have limited sensitivity to the picomolar range (i.e., 10^−12^ M) [33,34,35].

### 3.1. Neurofilament Light Chain (NfL)

NfL is an essential component of neurofilaments, which are cylindrical proteins exclusively residing within the neuronal cytoplasm [36,37]. Under physiological conditions, axons continuously release low levels of NfL in an age-dependent manner. However, Central Nervous System (CNS) axonal damage precipitates a marked increase in NfL release, which in turn enters the bloodstream through the BBB [37,38].

Blood NfL concentrations have been consistently found elevated in FTD patients (overall, bvFTD and PPA variants) compared to cognitively intact individuals [39,40,41,42,43,44,45,46,47,48,49,50]. One meta-analysis has particularly found that NfL levels in FTD are about 2.65 [1.59–4.43] times higher (ratio of means) compared to HC [39]. A second meta-analysis reported that NfL concentrations in FTD exceed those of HC by (mean difference—MD) 37.2 pg/mL [25.2–49.2] [40]. Another meta-analysis suggested that individuals with FTD exhibit increased NfL levels by an average of (standardized MD- SMD) 1.08 [0.72–1.43] standard deviations (SDs) [41]. A fourth meta-analysis showed that NfL levels in FTD are (SMD) 1.24 [1.01–1.48] SDs greater than HC [42].

Increased NfL levels were also documented among symptomatic mutation carriers (FTD cases) in relation to presymptomatic mutation carriers (*GRN*, *MAPT*, *C9orf72*) and asymptomatic first-degree non-carrier relatives [36,51,52,53,54,55]. Among genetically confirmed FTD cases, *GRN* mutation carriers usually exhibited higher NfL levels and faster rates of increase compared to other genetic subgroups (*MAPT*, *C9orf72*) [44,50,51,52,53,54,56,57].

Of note, NfL has been measured elevated in cases of prodromal FTD compared to HC [57,58,59], enhancing the diagnostic accuracy of the proposed criteria for prodromal disease (at a cut-off value of 8.53 pg/mL) [60]. Moreover, in prodromal stages and presymptomatic mutation carriers, higher baseline NfL concentrations have been associated with a greater hazard of progression to full-blown dementia and could discriminate converters from non-converters [36,53,55,57,58,59,61,62] (Table 2). Prognostic metrics have been reported to be increasingly superior closer to the conversion stage [62]. NfL levels have been found to escalate more conspicuously among converters, with a more abrupt increase over the conversion stage [53,55]. Intriguingly, higher NfL and steeper increases may also discriminate presymptomatic mutation carriers who progress to prodromal FTD from those who remain asymptomatic [59,62]

At the same time, baseline blood NfL levels in FTD have been correlated with more prominent (cross-sectionally) and accentuated (longitudinally) global and focal frontotemporal atrophy, worse cognitive performance, behavioral disturbances, and functional impairment, more abrupt cognitive, behavioral, and functional decline, as well as shorter survival [43,48,49,50,52,53,54,56,57,63,64,65,66,67,68]. Notably, these associations were substantiated for patients of different disease stages, even during prodromal FTD or among presymptomatic mutation carriers [53,54,57,61].

In parallel, some studies have found that FTD patients had higher Nfl levels than AD [43,47,56,66,67,69] and could even separate svPPA/nfvPPA from lvPPA [49,50,70]. On the contrary, others have revealed that blood NfL levels are only higher in certain FTD subgroups (e.g., nfvPPA) [44,48], have poor discriminatory potential [63], or have no difference from AD at all [45,46,71,72,73]. Higher levels of blood NfL were also found in FTD compared to DLB [47,64,71] and phenocopy FTD (non-progressive), allowing their differentiation [65]. Additionally, several studies have supported that increased NfL may distinguish FTD (mainly bvFTD) from PPDs (i.e., bipolar disorder, schizophrenia, depression) [66,74,75,76,77]. A detailed presentation of the studies examining the discriminatory properties (and proposing specific cut-offs) of NfL in FTD is in Table 3.

In summary, blood NfL levels constitute a significant biomarker of neurodegeneration. Although this biomarker may not be useful in the differentiation of FTD from AD, its utility in distinguishing bvFTD from PPDs or DLB remains promising. In cases of prodromal FTD and presymptomatic mutation carriers (*GRN*, *MAPT*, *C9orf72*, *TARDBP*), elevated NfL concentrations appear to be a harbinger of pheno-conversion to full-blown dementia. Baseline NfL levels correlate with steeper cognitive, behavioral and functional decline, as well as more rapid neuroanatomical changes, making NfL promising in monitoring disease progression. Nevertheless, these potential biomarker implications are far from applicable right now, since the standardization of processes and the establishment of reference intervals and/or thresholds require further work. Assay harmonization will enable comparisons between laboratories and ultimately facilitate the establishment of reference values.

### 3.2. Phosphorylated Neurofilament Heavy Chain (pNfH)

The primary source of pNfH is the axonal cytoskeleton. Its expression and phosphorylation determine axonal diameter, myelination, and conduction velocity. Following neuronal damage, neurofilaments are released into the interstitial fluid, CSF, and finally peripheral circulation, rendering blood pNfH an easily accessible blood biomarker [78,79].

pNfH levels have been thoroughly examined in various studies assessing MND patients. Fluid pNfH levels (CSF and blood) were elevated in ALS compared to HC and were inversely correlated with disease progression [76,79,80,81,82,83,84,85,86,87]. Fluid pNfH (CSF and blood) was also higher in ALS compared to FTD [79,88].

Research has shown that presymptomatic mutation carriers (*GRN*, *C9orf72*, or *MAPT*) and healthy non-carriers have similar blood pNfH levels [36,55]. Symptomatic (FTD) mutation carriers, however, exhibited greater pNfH than both groups—significant pNfH increases started proximally to the conversion stage [36,55]. Of note, one study did not report significant differences in pNfH among genetic subgroups (*C9orf72*, *GRN*, and *MAPT*) [89].

In summary, the small number of published studies on blood pNfH in FTD reflect a potential limited role in the demarcation of the conversion stage. Combined NfL and pNfH measurements may allow a wider dynamic stage-dependent stratification. However, elevated levels of pNfH are worth investigating in cases of ALS-FTD, considering the higher levels of pNfH among patients with ALS.

### 3.3. Tau

Tau is a protein encoded by the *MAPT* gene and is associated with microtubules in neurons, helping the stabilization of their internal structure. In several neurodegenerative diseases, including AD and some forms of FTD, tau proteins become abnormally aggregated and form neurofibrillary tangles. This aggregation disrupts normal cellular function and contributes to neurodegeneration [90]. Total tau is generally considered a marker of neuronal injury and neurodegeneration [91].

A few studies have shown that blood total tau does not differ between patients with FTD and HC [45,92,93]. On the other hand, there is also limited evidence that total tau is increased in FTD (in both behavioral and language variants) in relation to HC [63,94]. At the same time, similar levels of total tau were documented in cases with AD and FTD [45,63,92,93]. Additionally, there were no differences in total tau among clinical (behavioral and language) variants apart from ALS-FTD, which presented with significantly lower levels of total tau [92]. Moreover, genetic (*GRN*, *MAPT*, *C9orf72*) cases did not differ either from each other or from sporadic ones [92,94]. Finally, tau levels neither correlated with brain volumes in FTD nor predicted clinical decline or survival [63,92].

Overall, total tau blood levels appear to lack applicability in the framework of FTD.

### 3.4. Phosphorylated Tau (pTau)

Phosphorylation of tau is a normal regulatory process, but excessive phosphorylation can lead to the formation of pathological tau aggregates and, in turn, neurodegeneration [90,95]. The published literature has already shown that elevated blood p-tau levels in AD (compared to HC) have an important diagnostic value [96,97,98,99,100].

Previous research has also found that blood p-tau217 and p-tau181 levels are higher in AD compared to FTD, providing good discriminative properties (no consistent differences were found between FTD and HC) [32,43,44,47,64,67,69,101,102,103,104]. Moreover, higher p-tau levels have been specifically found in AD-associated lvPPA compared to FTD-related PPAs (nfvPPA, svPPA) [67,101].

With the exception of one study, similar p-tau levels have been reported between DLB and FTD [47,64,69]. No differences have been detected among clinical FTD subgroups (bvFTD, svFTD, nfvFTD, FTD plus), genetic subgroups (*GRN*, *MAPT*, *C9orf72*), and between genetic and sporadic cases [44,47,101,103]. At the same time, no correlations have been found between blood p-tau181 and structural indices, disease severity, cognitive function, behavioral measures, and survival for FTD patients [44,64,67].

Overall, p-tau is of potential value in the discrimination of AD from FTD (as well as of lvPPA from FTD-related PPAs) (Table 4).

### 3.5. Progranulin (PGRN)

Progranulin (PGRN) glycoprotein is encoded by 12 out of the 13 exons of the *GRN* gene on the chromosome 17q21, and it is an anti-inflammatory molecule with effects on various inflammatory, autoimmune diseases, and cancer [105,106]. Among the genetic causes of FTD are the *GRN* mutations, which lead to reduced levels of PGRN [107].

Published studies found that PGRN is an auspicious biomarker for detecting *GRN* mutation carriers. *GRN* mutations in symptomatic and presymptomatic carriers were linked to reduced PGRN levels in blood in relation to HC, presymptomatic carriers of other mutations (*C9orf72*, *MAPT*), and patients with FTD. [108,109,110,111,112,113,114,115,116,117]. Published research has not revealed any correlation between PGRN levels and brain volumes in FTD [117,118]. Of note, blood and CSF PGRN levels exhibit no to weak/moderate correlations [109,115,119,120,121,122]. These findings suggest some limitations in the use of this blood biomarker (e.g., non-*GRN* mutation carriers, sporadic cases, monitoring response to PGRN modulating agents).

In conclusion, PGRN concentrations could serve as a blood biomarker to identify *GRN* mutation carriers in asymptomatic or symptomatic individuals.

### 3.6. Glial Fibrillary Acidic Protein (GFAP)

The *GFAP* gene in locus 17q21 encodes the GFAP [123]. Glial fibrillary acidic protein (GFAP) is the main intermediate filament protein found in astrocytes, and it is responsible for structural integrity, cell movement, and shape change [36,124]. Its production is induced upon brain damage or degeneration and during normal aging due to astrocytosis [124].

Blood GFAP levels have been consistently found elevated in FTD (all phenotypic variants) compared to HC but not compared to AD [47,125,126,127,128,129]. More often, GFAP levels have been reported higher in those with AD in relation to individuals with FTD [47,64,128,130,131]. Of note, this difference has been occasionally found even from the pre-dementia stage, i.e., MCI-AD stage, with more pronounced elevations accompanying those with incident AD at follow-up [127,128,132].

On the other hand, published evidence does not suggest any differences in GFAP between FTD and other major neurocognitive entities (e.g., DLB, PSP, CBS), with occasional results in favor of higher GFAP levels in DLB [47,64,127]. Notably, cases with overlapping neuropathologies involving amyloid deposition exhibit higher GFAP levels relative to amyloid-negative cases [47]. With respect to PPDs, GFAP concentrations have been determined to increase in those with FTD and appear to possess optimal diagnostic properties [133].

Regarding genetic cases (*GRN*, *MAPT*, *C9orf72*), symptomatic *GRN* mutation carriers have higher GFAP levels compared to non-*GRN* cases [47,54,125]. Of note, one study revealed increased levels in presymptomatic *GRN* mutation carriers versus presymptomatic non-*GRN* mutation carriers [134].

From the clinical perspective, GFAP concentrations have been correlated negatively with cognitive performance, brain volumes, and functional independence and positively with rates of cognitive decline and temporal atrophy in those with FTD (AD as well) and presymptomatic *GRN* and *C9orf72* mutation carriers [54,125,126,127,130,133]. Moreover, high blood GFAP has been found to prelude incident FTD, although without specificity (AD and vascular dementia as well) [132,135].

Overall, blood GFAP appears to be a non-specific index of neurodegeneration, although amyloid pathology may present stronger associations compared to other neurodegenerative alterations. Nevertheless, its targeted capitalization may still assist in the differentiation of PPDs from bvFTD. It remains to be seen if GFAP may assume a role in the detection of asymptomatic or symptomatic *GRN* mutation carriers.

### 3.7. TAR DNA-Binding Protein-43 (TDP-43)

TDP-43, a ubiquitously expressed RNA/DNA-binding nuclear protein, is crucial in multiple cellular functions and has a pivotal role in the development of the central nervous system [136]. TDP-43 inclusions have been found in most cases of ALS and the most frequent form of familial and sporadic FTD, frontotemporal lobar degeneration with ubiquitinated inclusions [137,138], implying that TDP-43 is the common pathological substrate between FTLD-ALS [139]. Studies report that early, even presymptomatic loss of TDP-43 splicing repression (leading to detection of proteins containing cryptic exon-encoded epitopes) is an indicator of this underlying pathology [140,141,142].

Studies measuring blood TDP-43 levels demonstrated that FTD patients had higher concentrations compared to HC and other neurogenerative conditions, AD and PD [93,143,144,145]. Plasma extracellular vesicle TDP-43 levels have also been determined higher in ALS-FTD compared to HC [146]. In addition, TDP-43 levels were higher in *C9orf72* and *GRN* mutation carriers in relation to the remaining patients with FTD (*MAPT* mutation carriers or non-genetic cases) and HC [144]. Conversely, in one study, TDP-43 levels in the blood were significantly lower in the FTD group compared to the HC, a finding driven by *C9orf72* repeat expansion carriers [147]. Finally, a correlation has been determined between blood TDP-43 concentrations and the extent of phosphorylated TDP-43 brain changes in FTLD [148,149].

Although one study has generated contradictory results, TDP-43 appears to be a promising diagnostic blood biomarker. Its utility in terms of prognosis or other disease parameters is under debate. Finally, its potential in distinguishing TDP-43 pathology from other FTD-related pathologies requires further research.

### 3.8. Other Biomarkers and Interesting Insights

Apart from the evaluation of single biomarkers, their combination constitutes an intriguing prospect in the field of neurodegeneration [104]. Several studies have reported that the combination of CSF and/or blood biomarkers elevates the diagnostic accuracy in the evaluation of cognitively impaired individuals [69,126,127,150,151,152,153,154]. Subsequently, the use of a biomarkers’ panel could improve diagnostic metrics in FTD as well. Of note, the capitalization of clinical parameters (e.g., neurological findings, cognitive, neuropsychiatric, and motor symptoms) along with biomarkers could improve diagnostic metrics even more [43,71,155,156,157,158].

Other potential blood biomarkers include cytokines, chemokines, markers of lysosomal, synaptic and neurotransmitter function, and a variety of genetic markers [31,150,151]. Micro-RNAs are circulating short non-coding RNAs, and their differential expression may provide a discrete disease signature (for FTD, in general, or for particular genetic FTD cases in specific, e.g., cases with *C9orf72* mutations) [159]. Long non-coding RNAs, on the other hand, are longer molecules that are abundant in the CNS; however, their potential implications in clinical practice and research have been substantially less investigated [160]. Both molecules have demonstrated encouraging biomarker properties in the framework of FTD: for instance, miR-223–3p, miR-15a-5p, and miR-22–3p microRNA measurements in blood samples may distinguish FTD from AD [161], the relative expression of NORAD and NEAT1 long non-coding RNAs in mononuclear cells is more abundant in *C9orf72* carriers, and the relative transcription of GAS5 long non-coding RNAs is less abundant in mononuclear cells in *GRN* mutation carriers [162]; nevertheless, further research is warranted to establish their exact place in daily practice and research. Of note, polygenic risk scores combine data from genome-wide association studies to capture the individualized susceptibility towards FTD [163].

Lately, biomarker discovery strategies employ proteomics (quantification of alterations in protein levels of different biofluids), lipidomics (analysis of lipids in the extract of a biological sample), and metabolomics (study of metabolites and small molecules in biological specimens). These strategies could unravel a panel of biomarkers that include a combination of proteins, lipids, and metabolites specific to FTD [150].

## 4. Conclusions

In conclusion, the exploration of blood biomarkers presents a promising frontier in the quest for early and accurate diagnosis and prognosis of FTD. These biomarkers offer a less invasive, more tolerable, and potentially more cost-effective alternative to CSF and imaging biomarkers. We reviewed that, among the most rigorously studied blood biomarkers, NfL stands out for its ability to distinguish individuals at high risk of incident FTD as well as predict clinical and neuroanatomical progression. pTau may assist in differentiating FTD from AD. PGRN levels are particularly useful in identifying *GRN* mutation carriers; the role of GFAP in the detection of *GRN* mutation carriers is also under investigation. The latter may also serve as a biomarker of amyloid pathology. The role of TDP-43 in the FTD remains promising, as higher levels have been correlated to TDP-related pathological changes. Total tau and pNfH are of lesser value.

There is still much to explore with respect to blood biomarkers in FTD, so the conduction of well-designed and reported large multicenter studies will pave the way to fill in the gaps and wishfully enable the routine use of biomarkers in FTD. Particular focus should be placed on the harmonization of assays among different laboratories and the standardization of processes that will ultimately lead to the establishment of reference intervals and/or thresholds. The final step would be to reduce the cost of these biomarkers and make their widespread use eventually feasible.

New technologies have revolutionized biomarker research. To date, there is no single biomarker that can serve as a gold standard for the diagnosis of FTD. While individual biomarkers have shown some promise, the combination of multiple biological markers, along with imaging and genetic data, is likely to exhibit superior diagnostic and prognostic properties. In this direction, machine learning and artificial intelligence are increasingly capitalized on in an effort to create a ‘biomarker pattern’ that may allow the accurate diagnosis of FTD during the symptomatic and ideally the identification of undergoing FTD-related pathological alterations over the presymptomatic course. An even more ambitious endeavor would be to develop distinct ‘biomarker fingerprints’ for different subsets of individuals with FTD (e.g., patients with different underlying neurodegenerative changes) to ultimately pave the way towards precision medicine.

## Figures and Tables

**Table 1:** Blood-based biomarkers in frontotemporal dementia (FTD).

Biomarker	Primary Source	Primary Function	Process Implicated in Biomarker Change	Direction of Change and Potential Use
Neurofilament light chain (NfL) and Phosphorylated neurofilament heavy chain (pNfH)	Neuroaxonal cytoskeleton	Structural stability and radial growth of the neuroaxons	Neuroaxonal breakdown	Blood NfL concentrations have been consistently determined higher in FTD patients compared to healthy individuals. This biomarker may not serve the differential diagnosis of FTD from AD but may assist in the differentiation of bvFTD from PPDs or DLB. In prodromal FTD and presymptomatic mutation carriers (*GRN*, *MAPT*, *C9orf72*), increased levels of NfL (or steeper increases in serial measurements) increase the risk of incident FTD and may distinguish between converters and non-converters. Baseline NfL levels correlate with steeper cognitive, behavioral and functional decline, as well as more rapid neuroanatomical changes, making NfL promising in monitoring disease progression. Blood pNfH may have a potential limited role in the demarcation of the conversion stage to full-blown FTD.
Tau and Phosphorylated Tau (pTau)	Neuroaxonal cytoskeleton	Neuronal microtubule stabilization and axonal transport	Protein aggregation in a prion-like manner	Total tau levels may serve as a biomarker of neurodegeneration but probably lack applicability in the field of FTD. Higher p-tau concentrations may distinguish AD from FTD and logopenic variant PPA from FTD-related PPAs.
Progranulin (PGRN)	A subset of brain neurons and microglia	Regulates cell growth, lysosomal functions, inflammation, stress responses and neuronal survival.	Lysosomal dysfunction	PGRN could serve as a blood biomarker to identify *GRN* mutation carriers in asymptomatic or symptomatic individuals.
Glial fibrillary acidic protein (GFAP)	Astrocytes	Structural integrity, shape change and movement	Astrocytosis	GFAP exhibits stronger associations with amyloid pathology compared to other neurodegenerative alterations. Nevertheless, GFAP may assist in the differentiation of PPDs from bvFTD. GFAP may also assume a role in the detection of asymptomatic or symptomatic *GRN* mutation carriers.
TAR DNA-binding protein-43 (TDP-43)	Highly expressed in CNS progenitors for neuros and glia—in later stages it is expressed in differentiated neural cells as well—as the CNS matures expression diminishes	Regulates gene expression and RNA processing	Protein aggregation in a prion-like manner	TDP-43 appears to be a promising diagnostic blood biomarker and may distinguish TDP-43 pathology from other FTD-related pathologies.

PPA: primary progressive aphasia; PPDs: primary psychiatric disorders; DLB: dementia with Lewy bodies.

**Table 2:** Studies assessing the prognostic properties (AUC, specificity, sensitivity) of NfL in presymptomatic FTD mutation carriers (*GRN* and/or *C9orf72* and/or *MAPT* and/or *TARDBP*) in terms of conversion: from asymptomatic to prodromal and/or from asymptomatic and prodromal to full-blown FTD.

Author—Publication Year	Settings	Participants	Participant Characteristics, N (Female %), Age	Time to Conversion	NfL Thresholds	Prognostic Metrics
Giannini 2023	Erasmus University Medical Center (The Netherlands).	First-degree family members of patients with genetic FTD (*GRN*, *C9orf72*, *MAPT* or *TARDBP*). Participants who carry the genetic variant make up the presymptomatic or prodromal FTD group, i.e., at-risk for developing FTD.	Converters = 21 (71.4%), median age 55.7 years, IQR = 48.6–62.6 -Converted to prodromal FTD = 8 -Converted to full-blown FTD = 13 Non-converters = 61 (65.6%), median age 45.8 years, IQR = 39.2–55.0	Median 3 years (IQR = 1.8–5.0)	>3 years before conversion = 8.9 pg/mL 3–1.5 years before conversion = 12.3 pg/mL 1.5–0 years before conversion = 12.6 pg/mL 0–1.5 years after conversion = 14.2 pg/mL	>3 years before conversion -AUC = 0.860 -sensitivity = 80.0 -specificity = 77.1 3–1.5 years before conversion -AUC = 0.900 -sensitivity = 81.3 -specificity = 93.4 1.5–0 years before conversion -AUC = 0.920 -sensitivity = 90.9 -specificity = 95.1 0–1.5 years after conversion -AUC = 0.970 -sensitivity = 94.7 -specificity = 98.4
Gendron 2022	Multicenter study including participants from two North American multicenter observational studies (LEFFTDS/ARTFL).	The presymptomatic group consisted of individuals with a *C9orf72* repeat expansion or *GRN* or *MAPT* mutations, kindreds of adults with FTD-causing mutations. The HC group composed of clinically normal, mutation-negative individuals, kindreds of individuals with known FTD-causing mutations.	Presymptomatic mutation carriers = 85 (51.8%), median age 49, range = 40–71 HC = 144 (66.0%), median age 53, range = 40–80	Median 1.3 years (range: 1.0–2.8 years)	10 pg/mL	HC vs. presymptomatic -AUC = 0.64 HC vs. converters -AUC = 0.850 Non-converters vs. converters -AUC = 0.780
Wilke 2021	Participants from centers collaborating in the GENFI (Europe and Canada).	Healthy participants who carry the pathogenic FTD mutations (*GRN*, *MAPT* or *C9orf72*) make up the presymptomatic group.	Presymptomatic = 172 (63.0%), median age 41.2 years, IQR = 33.2–50.5 Converters = 7 (29.0%), median age 62.5 years, IQR = 52.2–65.6	Median 3.2 years	NR	NFL distinguished converters vs. non-converters -AUC = 0.910 NfL change rates converters vs. non-converters -AUC = 0.940
Van der Ende 2019	Participants from centers collaborating in the GENFI (Europe and Canada).	Healthy participants who carry the pathogenic FTD mutations (*GRN*, *MAPT* or *C9orf72*) make up the presymptomatic group.	Presymptomatic = 149 (65.0%), median age 45 years, IQR = 39–55 Converters = 9	NR	15 pg/mL	Converters vs. non-converters -AUC = 0.930 -sensitivity = 100 -specificity = 84.0

FTD: frontotemporal dementia; NfL: Neurofilament light chain; HC: Healthy controls; AUC: Area under the curve; IQR: interquartile range; NR: Not reported; N: number; GENFI: GENetic Frontotemporal dementia Initiative; LEFFTDS: Longitudinal Evaluation of Familial Frontotemporal Dementia Subjects; ARTFL: Advancing Research and Treatment for Frontotemporal Lobar Degeneration.

**Table 3:** Studies assessing the discriminatory properties (AUC, specificity, sensitivity) of NfL in FTD versus HC, AD, DLB and PPDs.

Author—Publication Year	Settings	Diagnostic Criteria	Participants per Group (Female %), Age in Years ± SD (Unless Stated Otherwise)	Disease Severity—CDR(-FTLD)	Time from Onset to Plasma Collection	NfL Thresholds	Diagnostic Metrics
Chen 2024	Memory clinics from four hospitals affiliated with the Zhejiang University School of Medicine (China).	Clinical diagnoses were supported by Aβ-Pet and CSF Aβ investigations.	bvFTD = 16 (62.5%), 64.3 ± 9.0 AD = 64 (48.4%), 62.8 ± 10.7 PSPci = 12 (58.3%), 71.8 ± 5.9 HC = 20 (50.0%), 65.7 ± 9.1	**Median CDR** bvFTD = 2.0 AD = 1.0 PSPci= 1.0	bvFTD = mean 3.6 years AD = mean 2.3 years PSPci = mean 2.5 years	bvFTD vs. HC = 17.3 pg/mL AD vs. bvFTD/PSPci = 55.0 pg/mL	bvFTD vs. HC -AUC = 0.946 -sensitivity = 91.7 -specificity = 95.0 AD vs. bvFTD/PSPci -AUC = 0.704 -sensitivity = 98.4 -specificity = 36.0
Benussi 2022	Centre for Neurodegenerative Disorders, Department of Clinical and Experimental Sciences, University of Brescia (Italy).	Clinical diagnoses were supported by brain structural Imaging. CSF concentrations of tau, p-Tau181, and Aβ were measured in a subset of cases. Genetic screening for *GRN*, *C9orf72*, and *MAPT* mutations was performed in familial cases and early-onset sporadic cases.	FTLD = 127 (47.8%), median age 64.0, IQR = 58.0–70.0 (67bvFTD, 44 PPA, 7 CBS, 9 PSP) AD = 48 (44.3%), median age 68.5, IQR = 61.8–73.0 HC = 27 (55.6%), median age 48.0, IQR = 38.0–68.0	**Median CDR-FTLD** FTLD = 4.0 AD = 2.0	FTLD= median 2 years, IQR = 1–3 AD= median 2 years, IQR = 1–3	NR	HC vs. disease groups -AUC = 0.940 FTLD vs. AD -AUC = 0.680
Gendron 2022	Multicenter study including participants from two North American multicenter observational studies (LEFFTDS/ARTFL).	Diagnoses were established clinically. The HC group composed of clinically normal, mutation-negative individuals, kindreds of individuals with known FTD-causing mutations.	bvFTD = 289 (41.2%), median age 62, range = 32–85 nfvPPA = 72 (54.2%), median age 70, range = 49–86 svPPA = 84 (48.8%), median age 66, range = 50–88 FTD-ALS = 25 (34.0%), median age 61, range = 45–75 HC = 144 (66.0%), median age 53, range = 40–80	**Median CDR-FTLD** The majority of: bvFTD = 1–2 nfvPPA = 0.5–2 svPPA = 0.5–2 FTD-ALS = 1–2	bvFTD = median 5 years, range = 0–32 nfvPPA= median 4 years, range = 1–12 svPPA = median 6 years, range = 1–34 FTD-ALS = median 3 years, range = 1–15	NR	bvFTD vs. HC -AUC = 0.920 nfvPPA vs. HC -AUC = 0.980 svPPA vs. HC -AUC = 0.980 FTD-ALS vs. HC -AUC = 0.970 **Cases with CDR-FTLD of 0.0 or 0.5** bvFTD vs. HC -AUC = 0.880 nfvPPA vs. HC -AUC = 0.940 svPPA vs. HC -AUC = 0.940
Baiardi 2022	Neuropathology Laboratory, Institute of Neurological Science of Bologna (Italy).	Clinical diagnoses were supported by neuroimaging, and CSF AD core biomarkers (tau, p-Tau181, and Aβ).	FTD = 59 (57.6%), 62.9 ± 8.9 AD = 97 (55.7%), 67.8 ± 9.3 PSP = 31 (35.5%), 69.2 ± 10.2 CBS = 29 (62.1%), 71.3 ± 7.2	**CDR score ≥ 1** FTD = 81.6% AD = 77.6% PSP = 54.8% CBS = 67.9% **CDR score ≥ 2** FTD = 41.9% AD = 38.8% PSP = 19.4% CBS = 17.9%	FTD = mean 34.3 months ± 33.5 AD = mean 41.7 months ± 34.9 PSP = mean 51.5 months ± 33.1 CBS = mean 43.2 months ± 37.4	FTD vs. other diseases = 31.3 pg/mL	FTD vs. other diseases (PSP/CBS/DLB/AD) -AUC = 0.761 -sensitivity = 72.9 -specificity = 74.3 FTD vs. AD -AUC = 0.791 FTD vs. DLB -AUC = 0.745
Chouliaras 2022	Memory clinics in and around Cambridgeshire and the North of England, the DNUK-CRN, and the JDRP.	Clinical diagnoses; PET-Aβ and MRI investigations were available for a subset of participants.	FTD = 28 (43.0%), 64.5 ± 8.6 MCI + AD = 63 (32.0%), 73.9 ± 7.8	NR	NR	MCI + AD vs. FTD = 0.58 in the log10 converted levels	MCI + AD vs. FTD -AUC = 0.850 -sensitivity = 89.0 -specificity = 75.0
Thijssen 2022	Study including participants from the Amsterdam Dementia Cohort (The Netherlands).	Clinical diagnoses were supported by electroencephalography, brain MRI, and CSF AD biomarker analysis. All patients with AD were CSF amyloid positive, and all controls were CSF amyloid negative.	**Cohort 1** FTD = 40 (50.0%), median age 64, IQR = 61–70 AD = 40 (50.0%), median age 58, IQR = 55–59 **Cohort 2** FTD = 38 (53.0%), median age 63, IQR = 59–67 AD = 38 (53.0%), median age 63, IQR = 59–67	NR	NR	NR	Cohort 1 FTD vs. AD -AUC = 0.790 -sensitivity = 48.0 -specificity = 98.0 Cohort 2 FTD vs. AD -AUC = 0.780 -sensitivity = 89.0 -specificity = 55.0
Thijssen 2021	Data collected from two cohorts; UCSF Memory and Aging Center (U.S.A.) and ARTFL (U.S.A. and Canada).	Clinical diagnoses were supported by brain MRI, biofluid collection and genetic testing. All clinically diagnosed amnestic AD patients, lvPPA and PCA, had biomarker confirmation with either Aβ-PET, autopsy or genetic biomarker. Genetic screening was conducted to identify mutations in the *C9orf72*, *GRN* and *MAPT* genes. Eighty-three participants from the UCSF Memory and Aging Center had a pathology-confirmed diagnosis.	AD = 58 (56.9%), 65 ± 10.0 lvPPA = 15 (53.3%), 63 ± 9.0 PCA = 2 (100.0%), 58 ± 11.0 CBC = 79 (43.0%), 67 ± 8.0 PSP = 74 (54.1%), 69 ± 7.0 bvFTD = 62 (40.3%), 61 ± 10.0 nfvPPA = 32 (46.9%), 70 ± 7.0 svPPA = 27 (59.3%), 70 ± 7.0	**Mean CDR -sb** AD = 6.0 lvPPA = 3.0 PCA = 2.0 CBC = 4.0 PSP = 4.0 bvFTD= 7.0 nfvPPA = 3.0 svPPA = 6.0	NR	**Clinical diagnostic groups**: FTLD vs. AD/lvPPA/PCA = 42.5 pg/mL **Autopsy confirmed cases:** AD vs. FTLD-tau/TDP = 34.4 pg/mL AD vs. FTLD-tau = 45.9 pg/mL AD vs. FTLD-TDP = 67.5 pg/mL	**Clinical diagnostic groups**: FTLD vs. AD/lvPPA/PCA -AUC = 0.820 -sensitivity = 82.0 -specificity = 74.0 **Autopsy confirmed cases:** AD vs. FTLD-tau/TDP -AUC = 0.970 -sensitivity = 93.0 -specificity = 100.0 AD vs. FTLD-tau -AUC = 0.970 -sensitivity = 90.0 -specificity = 100.0 AD vs. FTLD-TDP -AUC = 0.960 -sensitivity = 88.0 -specificity = 100.0
Rojas 2021	Patients were recruited through the North American multicenter observational studies (LEFFTDS and ARTFL: 19 research centers in total) and GENFI study, which involved 25 research centers across Europe and Canada.	**Original cohort** LEFFTDS enrolled members of families with a known *C9orf72*, *GRN*, or *MAPT* mutation. ARTFL enrolled participants who met research criteria for an FTLD syndrome and asymptomatic individuals with a family history of an FTLD syndrome. **Validation cohort** GENFI enrolled symptomatic carriers of *C9orf72*, *GRN*, or *MAPT* mutations and those at risk of carrying a mutation because (first-degree relatives).	**Original cohort** Asymptomatic carriers = 92 (53.3%), median age 44, range = 19–71 FTLD = 62 (61.3%), median age 61.5, range = 33–74 MBI/MCI = 33 (45.5%), median age 54, range = 19–50 Asymptomatic non-carriers = 90 (64.4%), median age 50, range = 24–76 **Validation cohort** Asymptomatic carriers = 115 (63.5%), median age 41, range = 20–73 FTLD = 51 (45.1%), median age 63, range = 39–78 MBI/MCI = 32 (43.8%), median age 52, range = 29–75 Asymptomatic non-carriers = 99 (51.5%), median age 41, range = 20–73	**Median CDR-FTLD -sb** **Original cohort** Asymptomatic carriers = 0.0 FTLD =7.2 MBI/MCI = 1.5 Asymptomatic non-carriers = 0 ± 0.0 **Validation cohort** Asymptomatic carriers = 0.0 FTLD = 10.5 MBI/MCI = 1.0 Asymptomatic non-carriers = 0.0	NR	**Original cohort** FTLD vs. asymptomatic/MCI/MBI= 13.6 pg/mL **Validation cohort** FTLD vs. asymptomatic/MCI/MBI = 19.8 pg/mL	**Original cohort** FTLD vs. asymptomatic/MCI/MBI -AUC = 0.901 -sensitivity = 87.5 -specificity = 82.7 Asymptomatic vs. MCI/MBI -AUC = 0.676 FTLD vs. MCI/MBI -AUC = 0.803 **Validation cohort** FTLD vs. asymptomatic/MCI/MBI -AUC = 0.907 -sensitivity = 8.74 -specificity = 8.43 Asymptomatic vs. MCI/MBI -AUC = 0.641 FTLD vs. MCI/MBI -AUC = 0.805
Illán-Gala 2021	Memory and Aging Center, UCSF (USA).	Diagnosed at a multidisciplinary consensus conference according to clinical criteria. Clinicians were blinded to biomarker results.	FLTD (bvFTD, nfvPPA, svPPA, PSP, CBS) = 167 (50.3%), 65.8 ± 8.0 AD = 43 (62.8%), 65.2 ± 10.0 HC = 55 (54.5%), 52.2 ± 13.0	**Mean CDR-FTLD -sb** FTLD = 6.8 AD = 6.6 HC = 0.0	NR	NR	FTLD vs. HC AUC = 0.970 FTLD vs. AD AUC = 0.750
Benussi 2020	Data from two cohorts: Centre for Neurodegenerative Disorders, University of Brescia (Italy) and IRCCS Istituto San Giovanni di Dio Fatebenefratelli, Brescia (Italy).	Clinical diagnoses were supported by brain structural imaging while CSF biomarkers (Aβ, p-tau 181, total tau) were measured in a subset of cases. Genetic screening for *GRN*, *C9orf72*, and *MAPT* mutations was performed in familial cases and early-onset sporadic cases.	bvFTD = 134 (58.2%), 64.5 ± 8.0 avPPA = 48 (43.8%), 67.7 ± 8.8 svPPA = 27 (59.3%), 64.0 ± 8.2 CBS = 51 (52.9%), 65.8 ± 7.6 PSP = 31 (51.6%), 72.9 ± 7.4 AD = 63 (31.7%), 75.5 ± 8.1 HC = 63 (20.6%), 65.4 ±12.1	**Mean CDR-FTLD -sb** bvFTD = 7.9 avPPA = 6.2 svPPA = 5.7 CBS = 4.3 PSP = 4.2 AD = NR	bvFTD = mean 2.9 years ± SD = 2.8 avPPA = mean 2.8 years ± SD = 2.6 svPPA = mean 3.3 years ± SD = 2.2 CBS = mean 2.5 years ± SD = 1.8 PSP = mean 4.1 years ± SD = 2.8 AD = mean 1.5 years ± SD = 1.7	FTLD vs. HC = 22.5 pg/mL mild FTLD vs. HC = 18.0 pg/mL	FTLD vs. HC -AUC = 0.862 -sensitivity = 71.5 -specificity = 92.1 mild FTLD (CDR-FTLD ≤ 6.5) vs. HC -AUC = 0.808 -sensitivity = 74.8 -specificity = 74.2
Matías-Guiu 2019	Spain, exact settings were NR.	Clinical diagnoses were supported by FDG-PET studies.	lvPPA = 16 (37.5%), 73.81 ± 7.6 svPPA = 12 (33.3%), 74.83 ± 9.0 nfvPPA = 13 (61.5%), 71.31 ± 8.2 HC = 13 (23.1%), 75.08 ± 6.7	**Mean CDR** lvPPA = 1.1 svPPA = 2.0 nfvPPA = 1.2 bvFTD = 2.2 HC = 0.0 **Mean CDR-FTLD -sb** lvPPA = 6.53 svPPA = 13.3 nfvPPA = 7.8 bvFTD = 14.3 HC = 0.0	NR	NR	PPA vs. HC -AUC = 0.919
Steinacker 2018	Participants from the German FTLD consortium (Germany).	Diagnoses were established clinically.	bvFTD = 74 (40.5%), 63.7 ± 9.2 AD = 26 (42.3%), 67 ± 8.1 HC = 15 (60.0%), 64.8 ± 11.3	**Mean CDR -sb** bvFTD = 6.5 AD = 5.1 HC = 0.1 **Mean CDR-FTLD -sb** bvFTD = 8.8 AD = 6.6 HC = 0.1	bvFTD = mean 3.9 years ± 3.4 AD = mean 3.4 years ± 2.1	bvFTD vs. HC = 19.5 pg/mL bvFTD vs. AD = 29.5 pg/mL	bvFTD vs. HC -AUC = 0.851 -sensitivity = 91.0 -specificity = 79.0 bvFTD vs. AD -AUC = 0.676 -sensitivity = 74.0 -specificity = 58.0
Steinacker 2017	Participants from the German FTLD consortium (Germany).	Clinical diagnoses were supported by imaging studies.	nfvPPA + svPPA = 78 (52.6%), median age 65.3 years, range = 45–80 lvPPA = 21 (38.1%), median age 68.6 years, range = 49–78 HC = 35 (54.3%), median age 63.6 years, range = 37–75	**Median CDR -sb** nfvPPA + svPPA = 2.5 lvPPA = 2.8 **Median FTLD-CDR -sb** nfvPPA + svPPA = 4.5 lvPPA = 5.0	nfvPPA + svPPA = median 2.6 years, range = 0.2–19.9 lvPPA = median 3.3 years, range = 0.5–17.7	nfvPPA + svPPA vs. HC = 25 pg/mL nfvPPA + svPPA vs. lvPPA = 31 pg/mL	nfvPPA + svPPA vs. HC -AUC = 0.845 -sensitivity = 95.0 -specificity = 70.0 nfvPPA + svPPA vs. lvPPA -AUC = 0.767 -sensitivity = 81.0 -specificity = 67.0
Rohrer 2016	Participants from the University College London FTD study.	Diagnoses were established clinically. Participants were tested for *GRN*, *MAPT* or *C9orf72* mutations.	FTD = 67 (38.8%), 64.5 ± 7.9 (34bvFTD, 3 FTD-MND, 13 nfvFTD, 10 svFTD, and 10 PPA-not otherwise specified) HC = 28 (53.6%), 63.9 ± 7.2	NR	FTD = mean 5.5 years ± 3.7	FTD vs. HC = 33 pg/mL	FTD vs. HC -sensitivity = 84.0 -specificity = 96.0
Meeter 2016	Participants from 11 centers collaborating in the GENFI (Europe and Canada).	FTD was clinically diagnosed in patients with pathogenic mutations in *GRN*, *MAPT* or *C9orf72*. Healthy participants who carry the genetic variant make up the presymptomatic group. Cognitively healthy subjects without a pathogenic mutation make up the HC group.	FTD = 101 (51.0%), median age 59 years, IQR = 56–65 Presymptomatic carriers = 62 (63.0%), median age 49 years, IQR = 42–57 HC = 71 (59.0%), median age 54 years, IQR = 43–61	NR	FTD = median 2.0 years, IQR = 1.3–3.4	FTD vs. HC = 9.3 pg/mL Presymptomatic carriers vs. HC = 8.3 pg/mL	FTD vs. HC -AUC = 0.970 -sensitivity = 91.0 -specificity = 100 Presymptomatic carriers vs. HC -AUC = 0.630 -sensitivity = 34.0 -specificity = 97.0

SD: Standard deviation; NfL: Neurofilament light chain; AUC: Area under the curve; IQR: interquartile range; NR: Not reported; PET: positron emission tomography; MRI: Magnetic Resonance Imaging; CSF: cerebrospinal fluid; (a)AD: (amnestic) Alzheimer’s disease; DSM: Diagnostic and Statistical Manual of Mental Disorders; NIA-AA: National Institute on Aging—Alzheimer’s Association; (bv)FTD: (Behavioral variant) Frontotemporal dementia; PSP(ci): Progressive supranuclear palsy (with cognitive impairment); FTLD: Frontotemporal lobar degeneration; (nfv; sv; av; lv)PPA: (Non-fluent variant; semantic variant; agrammatic variant; logopenic variant) Primary progressive aphasia; ALS: Amyotrophic lateral sclerosis; DLB: Dementia with Lewy bodies; CBS: Corticobasal syndrome; MND: motor neuron disease; PCA: Posterior cortical atrophy; MBI/MCI: Mild Behavioral Impairment / Mild cognitive impairment; HC: Healthy controls; CDR (-FTLD) -sb: Clinical Dementia Rating dementia staging instrument (-plus National Alzheimer’s Coordinating Center behavior and language domains) -sum of boxes; GENFI: GENetic Frontotemporal dementia Initiative; LEFFTDS: Longitudinal Evaluation of Familial Frontotemporal Dementia Subjects; ARTFL: Advancing Research and Treatment for Frontotemporal Lobar Degeneration; UCSF: University of California, San Francisco; DNUK-CRN: Dementia and Neurodegeneration specialty of the UK Clinical Research Network; JDRP: Join Dementia Research platform.

**Table 4:** Studies assessing the discriminatory properties (AUC, specificity, sensitivity) of p-tau in FTD versus AD.

Author—Year	Country-Settings	Diagnosis	Sample (Female %), Age in Years ± SD (unless Stated Otherwise Specified)	CDR (Plus NACC FTLD)	Time from Onset to Plasma Collection	P-tau 181 Thresholds (unless P-tau 217 Values Are Specified)	Diagnostic Metrics
Benussi 2022	Centre for Neurodegenerative Disorders, Department of Clinical and Experimental Sciences, University of Brescia (Italy).	Clinical diagnoses were supported by brain structural imaging. CSF concentrations of tau, p-Tau181, and Aβ were measured in a subset of cases. Genetic screening for *GRN*, *C9orf72*, and *MAPT* mutations was performed in familial cases and early-onset sporadic cases.	FTLD = 127 (47.8%), median 64.0 years, IQR = 58.0–70.0 (67bvFTD, 44 PPA, 7 CBS, 9 PSP) AD = 48 (44.3%), median 68.5 years, IQR = 61.8–73.0	**Median CDR- FTLD** FTLD = 4.0 AD = 2.0	FTLD= median 2 years, IQR = 1–3 AD = median 2 years, IQR = 1–3	NR	FLTD vs. AD -AUC = 0.700
Baiardi 2022	Neuropathology Laboratory, Institute of Neurological Science of Bologna (Italy).	Clinical diagnoses were supported by neuroimaging, and CSF AD core biomarkers (tau, p-Tau181, and Aβ).	FTD = 59 (57.6%), 62.9 ± 8.9 AD = 97 (55.7%), 67.8 ± 9.3 PSP = 31 (35.5%), 69.2 ± 10.2 CBS = 29 (62.1%), 71.3 ± 7.2	**% CDR score ≥ 1** FTD = 82 AD = 78 PSP = 55 CBS = 68 **% CDR score ≥ 2** FTD = 42 AD = 39 PSP = 19 CBS = 18	FTD = mean 34.3 months ± 33.5 AD = mean 41.7 months ± 34.9 PSP = mean 51.5 months ± 33.1 CBS = mean 43.2 months ± 37.4	AD vs. other diseases = 1.98 pg/mL	AD vs. PSP/CBS/DLB/FTD -AUC = 0.889 -sensitivity = 86.6 -specificity = 80.0 FTD vs. AD -AUC = 0.964
Chouliaras 2022	Patients from specialist memory clinics in and around Cambridgeshire and the North of England, the DNUK-CRN, and the JDRP.	Clinical diagnoses; PET-Aβ and MRI investigations were available for a subset of participants.	FTD = 28 (43.0%), 64.5 ± 8.6 MCI + AD = 63 (32.0%), 73.9 ± 7.8	NR	NR	MCI + AD vs. FTD = 0.65 in log10 converted levels	FTD vs. MCI + AD -AUC= 0.88 -sensitivity = 85.0 -specificity = 79.0
Thijssen 2022	Study including participants from the Amsterdam Dementia Cohort (the Netherlands).	Clinical diagnoses were supported by electroencephalography, brain MRI, and CSF AD biomarker analysis. All patients with AD were CSF amyloid positive, and all controls were CSF amyloid negative.	**Cohort 1** FTD = 40 (50.0%), median age 64 years, IQR = 61–70 AD = 40 (50.0%), median age 58 years, IQR = 55–59 **Cohort 2** FTD = 38 (53.0%), median age 63 years, IQR = 59–67 AD = 38 (53.0%), median age 63 years, IQR = 59–67	NR	NR	NR	Cohort 1 FTD vs. AD -AUC = 0.850 -sensitivity = 74.0 -specificity = 97.0 Cohort 2 FTD vs. AD -AUC = 0.710 -sensitivity = 66.0 -specificity = 76.0
Thijssen 2021	Data collected from two cohorts; UCSF Memory and Aging Center (U.S.A.) and ARTFL (U.S.A. and Canada).	Clinical diagnoses were supported by brain MRI, biofluid collection and genetic testing. All clinically diagnosed amnestic AD patients, lvPPA and PCA, had biomarker confirmation with either Aβ-PET, autopsy or genetic biomarker. Genetic screening was conducted to identify FTLD-causing mutations in the *C9orf72*, *GRN* and *MAPT* genes. Eighty-three participants from the UCSF Memory and Aging Center had a pathology-confirmed diagnosis.	AD = 58 (56.9%), 65 ± 10.0 lvPPA = 15 (53.3%), 63 ± 9.0 PCA = 2 (100.0%), 58 ± 11.0 CBC = 79 (43.0%), 67 ± 8.0 PSP = 74 (54.1%), 69 ± 7.0 bvFTD = 62 (40.3%), 61 ± 10.0 nfvPPA = 32 (46.9%), 70 ± 7.0 svPPA = 27 (59.3%), 70 ± 7.0	**Mean CDR -sb** AD = 6.0 lvPPA = 3.0 PCA = 2.0 CBC = 4.0 PSP = 4.0 bvFTD= 7.0 nfvPPA = 3.0 svPPA = 6.0	NR	**Clinical diagnostic groups:** FTLD vs. AD/lvPPA/PCA p-tau217 = 0.19 pg/mL p-tau181 = 0.99 pg/mL **Autopsy confirmed cases:** AD vs. FTLD-tau/TDP p-tau217 = 0.17 pg/mL p-tau181 = 0.90 pg/mL AD vs. FTLD-tau p-tau217 = 0.18 pg/mL p-tau181 = 0.91 pg/mL AD vs. FTLD-TDP p-tau217 = 0.13 pg/mL p-tau181 = 0.77 pg/ml	**Clinical diagnostic groups:** FTLD vs. AD/lvPPA/PCA p-tau217 -AUC = 0.930 -sensitivity = 97.0 -specificity = 82.0 p-tau181 -AUC = 0.910 -sensitivity = 96.0 -specificity = 81.0 **Autopsy confirmed cases**: AD vs. FTLD-tau/TDP p-tau217 -AUC = 0.960 -sensitivity = 87.0 -specificity = 100.0 p-tau181 -AUC = 0.910 -sensitivity = 85.0 -specificity = 93.0 AD vs. FTLD-tau p-tau217 -AUC = 0.960 -sensitivity = 87.0 -specificity = 100.0 p-tau181 -AUC = 0.900 -sensitivity = 83.0 -specificity = 93.0 AD vs. FTLD-TDP p-tau217 -AUC = 0.980 -sensitivity = 94.0 -specificity = 93.0 p-tau181 -AUC = 0.950 -sensitivity = 94.0 -specificity = 93.0
Benussi 2020	Data from two cohorts: Centre for Neurodegenerative Disorders, University of Brescia (Italy) and IRCCS Istituto San Giovanni di Dio Fatebenefratelli, Brescia (Italy).	Clinical diagnoses were supported by brain structural imaging while CSF biomarkers (Aβ, p-tau 181, total tau) were measured in a subset of cases. Genetic screening for *GRN*, *C9orf72*, and *MAPT* mutations was performed in familial cases and early-onset sporadic cases.	bvFTD = 134 (58.2%), 64.5 ± 8.0 avPPA = 48 (43.8%), 67.7 ± 8.8 svPPA = 27 (59.3%), 64.0 ± 8.2 CBS = 51 (52.9%), 65.8 ± 7.6 PSP = 31 (51.6%), 72.9 ± 7.4 AD = 63 (31.7%), 75.5 ± 8.1	**Mean CDR (-FTLD) -sb** bvFTD = 7.9 avPPA = 6.2 svPPA = 5.7 CBS = 4.3 PSP = 4.2 AD = NR	bvFTD = mean 2.9 years ± 2.8 avPPA = mean 2.8 years ± 2.6 svPPA = mean 3.3 years ± 2.2 CBS = mean 2.5 years ± 1.8 PSP = mean 4.1 years ± 2.8 AD = mean 1.5 years ± 1.7	FTLD vs. AD = 5.88 pg/mL	FTLD vs. AD -AUC = 0.930 -sensitivity = 81.4 - specificity = 93.5 Mild FTLD (CDR ≤ 6.5) vs. mild AD (MMSE ≥ 20) -AUC = 0.909 -sensitivity = 89.3 - specificity = 82.0
Karikari 2020	Data from the TRIAD (McGill University, Canada) and BioFINDER-2 (Lund University, Sweeden) cohorts.	Clinical diagnoses were supported by CSF (Aβ, p-tau 181, total tau) and PET (tau, Aβ) biomarkers.	**TRIAD:** AD = 33 (45.0%), 64.6 ± 9.2 FTD = 8 (88.0%), 59.3 ± 8.5 **BioFINDER-2**: AD = 126 (53%), 74.0 ± 6.9 FTD = 18 (72%), 67.4 ± 7.4	NR	NR	NR	**TRIAD**: FTD vs. AD AUC = 1.000 **BioFINDER-2**: FTD vs. AD AUC = 0.828
Thijssen 2020	Participants from three cohorts, including the UCSF Memory and Aging Center (U.S.A.), the ARTFL consortium (U.S.A. and Canada) and Eli Lilly sponsored research study (U.S.A.).	Clinical diagnoses were supported by biomarkers or post-mortem pathological investigations in the vast majority of cases. Aβ-PET was available in 226 participants, 138 had tau-PET, 220 participants had MRI, 74 had previous CSF pTau181 concentrations available, 76 were carriers of FTLD-causing mutations (*GRN*, *C9orf72*, and *MAPT*) and 82 cases had an autopsy-confirmed diagnosis. All AD patients had either Aβ-PET, MRI, autopsy or genetic biomarker verification.	AD = 56 (58.9%), 65.0 ± 9.0 CBS = 39 (59.0%), 68.0 ± 8.0 PSP = 48 (56.3%), 69.4 ± 7.0 bvFTD = 50 (44.0%), 58.3 ± 9.0 nfvPPA = 27 (44.4%), 70.5 ± 7.0 svPPA = 26 (61.5%), 69.3 ± 7.0 MCI = 47 (44.7%), 60.8 ± 14.0	**Mean CDR -sb** AD = 4.8 CBS = 3.3 PSP = 4.7 bvFTD = 7.8 nfvPPA = 3.4 svPPA = 6.0 MCI = 2.0	AD = mean 6.0 years ± 3.0 CBS = mean 6.0 years ± 4.0 PSP = mean 6.7 years ± 3.0 bvFTD = mean 8.5 years ± 8.0 nfvPPA = mean 5.9 years ± 2.0 svPPA = mean 8.0 years ± 4.0 MCI = mean 5.7 years ± 3.0	**Clinical diagnostic groups:** AD vs. FTLD = 8.7 pg/mL **Autopsy confirmed cases:** AD vs. FTLD-tau/TDP = 9.5 pg/mL AD vs. FTLD-tau = 9.6 pg/mL AD vs. FTLD-TDP = 9.4 pg/mL FTLD-TDP vs. FTLD-tau = 9.6 pg/mL	**Clinical diagnostic groups:** FTLD vs. AD -AUC = 0.894 - sensitivity = 98.2 -specificity = 71.1 **Autopsy confirmed cases:** AD vs. FTLD-tau/TDP -AUC = 0.878 - sensitivity = 100.0 -specificity = 67.2 AD vs. FTLD-tau -AUC = 0.858 - sensitivity = 100.0 -specificity = 63.5 AD vs. FTLD-TDP -AUC = 0.947 - sensitivity = 100.0 -specificity = 80.0 FTLD-tau vs. FTLD-TDP -AUC = 0.664 - sensitivity = 98.1 -specificity = 33.3

SD: standard deviation; IQR: interquartile range; AUC: area under the curve; NR: not reported; CSF: cerebrospinal fluid; PET: positron emission tomography; MRI: magnetic resonance imaging; CBS: corticobasal syndrome; PSP: progressive supranuclear palsy; (bv) FTD: (behavioral variant) frontotemporal dementia; (lv; nfv; sv; av) PPA: (logopenic variant; non-fluent variant; semantic variant; agrammatic variant) primary progressive aphasia; FTLD: frontotemporal lobar degeneration; MND: motor neuron disease; AD: Alzheimer’s disease; PCA: posterior cortical atrophy; MCI: mild cognitive impairment; DLB: dementia with Lewy bodies; CDR (-FTLD) -sb: Clinical Dementia Rating dementia staging instrument (-plus National Alzheimer’s Coordinating Center behavior and language domains) -sum of boxes; TDP: TAR binding protein; DNUK-CRN: Dementia and Neurodegeneration specialty of the UK Clinical Research Network; JDRP: Join Dementia Research platform.

## Data Availability

Not applicable.
